# Dioxin Emissions and Human Exposure in China: A Brief History of Policy and Research

**DOI:** 10.1289/ehp.1103535

**Published:** 2011-03

**Authors:** Bin Zhao, Minghui Zheng, Guibin Jiang

**Affiliations:** State Key Laboratory of Environmental Chemistry and Ecotoxicology, Research Center for Eco-environmental Sciences, Chinese Academy of Sciences, Beijing, China, E-mail: binzhao@rcees.ac.cn

In October 2010, nine ministries and commissions of China jointly issued *Guidance on the Strengthening of Dioxin Pollution Prevention* [Ministry of Environmental Protection of People’s Republic of China (MEP) 2010], which requires key dioxin-emitting industries to carry out comprehensive actions to reduce dioxin emissions. This is a major historical milestone in China’s fight against dioxin pollution. China is one of the largest dioxin-emitting counties in the world, with total annual dioxin emissions from various sources estimated at 10 kg toxic equivalent (TEQ), about half of which is emissions to the atmosphere (Stockholm Convention on Persistent Organic Pollutants 2008). However, efforts to control dioxin pollution in China began later than in developed countries.

In 2000, China issued the first dioxin emission standard for waste incineration, which has been the main focus of dioxin pollution control in China. However, the focus of dioxin control in China is now being widely extended to include other key dioxin-emitting industries, such as ferrous and secondary nonferrous metal production. In addition, the new guidance document aims to establish a dioxin pollution control system and a mechanism for long-term supervision. Reduction, elimination, and prevention of dioxin pollution will take time, but China has taken action to address these issues with the release of this guidance document. It is a remarkable step forward.

China’s economy has been developing with dramatic speed in the past several decades, but environment-related research—including research on dioxin—has lagged far behind. In the late 1980s, research focused mainly on analysis of dioxin by-product residues resulting from the production of pentachlorophenol (PCP) and its sodium salt, and it wasn’t until 1996 that China established the nation’s first dioxin analysis laboratory in Wuhan. However, China has increasingly recognized the importance of science and technology in solving problems of environmental pollution and has supported a number of basic research projects on dioxin and other persistent organic pollutants (Jiang 2010). Since 2002, capacity building has greatly accelerated, and China currently has nearly 30 fully equipped dioxin analysis laboratories. Utilizing these facilities and the support of national basic research grants, Chinese scientists have conducted extensive studies on factors such as background levels, distribution, transfer, and transformation patterns of dioxin in the environment. These studies have provided an unprecedented fundamental picture of dioxin pollution across China.

In addition to carrying out environmental pollution monitoring and inspection of imported and exported products, some laboratories have performed pilot studies on dioxin exposure in the Chinese population. Li et al. (2009) reported that the range of upper-bound total TEQ in human milk samples was 2.59–9.92 pg TEQ/g lipid, with significantly lower levels in samples from rural versus urban areas. They also found positive correlations between the total TEQ level in human milk and consumption of aquatic food and meat, suggesting that diet is the main dioxin exposure pathway in the Chinese population, consistent with findings from the United States and Europe. However, the level of dioxin exposure in the Chinese population appears to be lower than in other countries (Li et al. 2009; Tanabe and Kunisue 2007). For example, in a study population from Shenzhen China, the estimated monthly intake of dioxin based on food intake profiles was 40.9 pg World Health Organization (WHO)-TEQ/kg body weight (Zhang et al. 2008).

Historic use of PCP and sodium pentachlorophenate (Na-PCP)—and probably other pesticides—seems to be an important source of dioxin in contemporary breast milk samples. Xiao et al. (2010) estimated dioxin body burdens in childbearing women and the general population of the Dongting Lake area of China, where Na-PCP has been used to control the spread of snail-borne schistosomiasis since the 1960s. The authors reported a positive correlation between blood dioxin levels and age, in agreement with most other studies. Although dioxin levels in breast milk were relatively low, exposure during the breast-feeding period is still a matter of concern.

Another area of concern is electronic waste (e-waste) disposal and processing in China, which exposes local populations to a complicated mixture of pollutants, including dioxin. One human exposure assessment (Chan et al. 2007) indicated that biologic samples from study participants living near e-waste processing sites had significantly higher levels of dioxin [human milk, 21.02 pg WHO-TEQ/g fat; placenta, 31.15 pg WHO-TEQ/g fat] than samples from other participants. In addition, the estimated daily intake of dioxin from birth to 6 months of age among infants breast-fed by mothers who worked at e-waste processing sites was two times higher than the estimated intake among infants at the reference site. Zhang et al. (2010) reported that pregnant women in an e-waste recycling town had significantly lower thyroid hormone levels than pregnant women in a reference town, and that total thyroxine levels were negatively correlated with body burdens of dioxin. These findings suggest that inappropriate e-waste recycling operations are responsible for increased dioxin levels in the local environment and in humans and that these exposures also may have health implications for the next generation.

The relationship between dioxin exposure and the health of the Chinese population is far from clear, in part because of the historical focus of basic research on environmental pollutant levels rather than on environmental health. In addition, China does not yet have an environmental health risk assessment system based on specific dioxin emission sources and population exposure patterns. Environmental health has become an increasingly high priority globally, and it is important to further encourage and enhance basic environmental health research in China in order to provide a scientific basis for environmental policies to protect the environment, ecological systems, and human health.

Most recently, increased construction of municipal solid waste incinerators has raised concerns about the release of dioxin during waste incineration and potential adverse health effects on local populations. In response, a number of research projects to evaluate health effects in communities living near incineration plants have been launched. With increasing public concern and the growing emphasis on controlling dioxin emissions by China, there is reason to hope and expect that China’s response to its dioxin pollution issues will continue to expand.

## Figures and Tables

**Figure:**
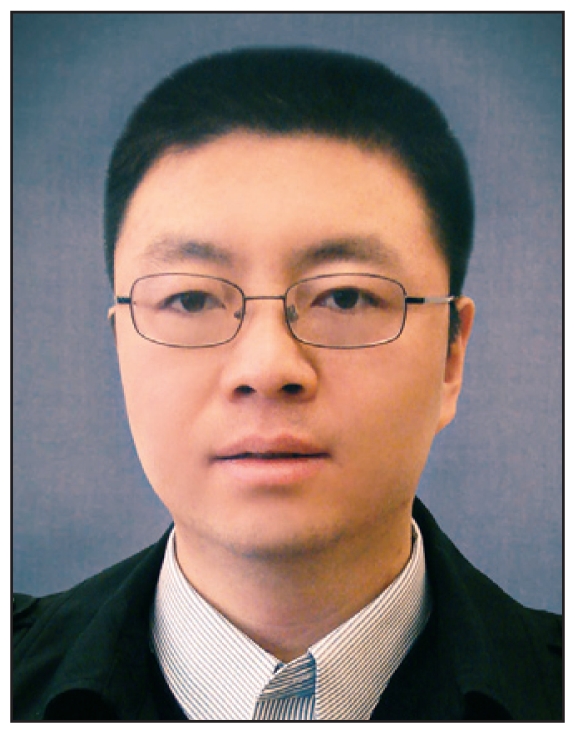
Bin Zhao

**Figure:**
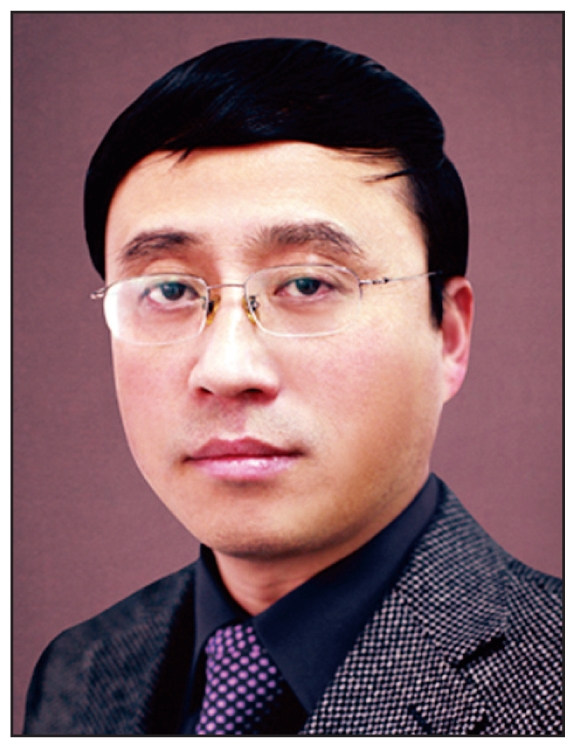
Minghui Zheng

**Figure:**
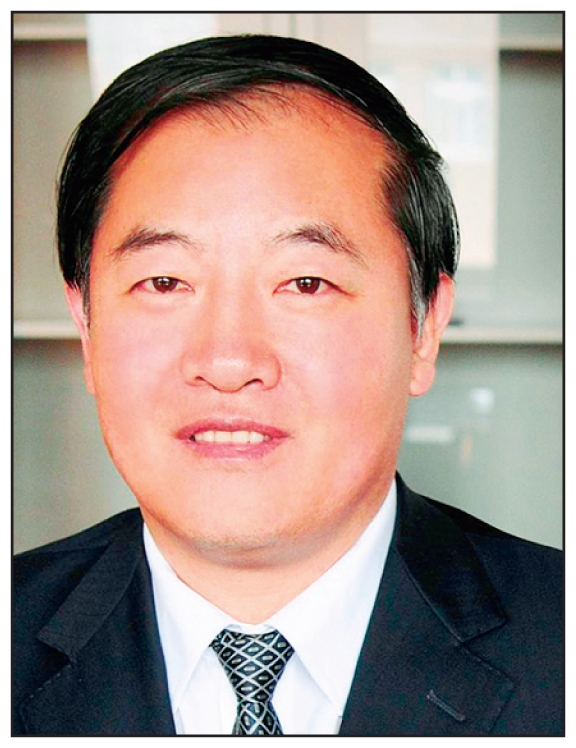
Guibin Jiang
